# Comparison of chemotherapy regimens plus rituximab in adult Burkitt lymphoma: A single-arm meta-analysis

**DOI:** 10.3389/fonc.2022.1063689

**Published:** 2022-12-23

**Authors:** Xiaoxuan Lu, Yu Liu, Ruyu Liu, Jiaxin Liu, Xiaojing Yan, Liren Qian

**Affiliations:** ^1^ Senior Department of Hematology, The Fifth Medical Center of People's Liberation Army (PLA), General Hospital, Beijing, China; ^2^ Department of Hematology, The Sixth Medical Center of People's Liberation Army (PLA), General Hospital, Beijing, China; ^3^ Department of Hematology, The First Affiliated Hospital of China Medical University, Shenyang, China

**Keywords:** Burkitt lymphoma, meta-analysis, chemotherapy regimen, rituximab, single-arm

## Abstract

**Background and aim:**

Given the paucity of evidence-based treatment recommendations, the most appropriate first-line regimen for adult Burkitt lymphoma is currently undefined. We aimed to identify the optimal treatment regimen containing rituximab for adult Burkitt lymphoma patients.

**Methods:**

The PubMed, Embase, Web of Science, and Cochrane databases were searched in December 2021 (10). We included all studies for the treatment of Burkitt lymphoma including rituximab. We excluded studies of patients aged ≤14 years old and those with sample numbers ≤10 patients. Random-effects models were used to compare different chemotherapy regimens regarding estimated 2-year overall survival (OS) rate, 2-year progression-free survival (PFS) rate, and overall response rate (ORR).

**Results:**

A total of 17 studies were included in this meta-analysis and divided into four groups: CODOX-M/IVAC, DA-EPOCH, GMALL-B-ALL/NHL2002, and Hyper-CVAD. DA-EPOCH was associated with a significantly higher 2-year OS rate [0.95, 95% confidence interval (CI) 0.86–1.00]. There was no significant difference in the 2-year PFS rates (0.81, 95% CI 0.76–0.85) and ORR (0.90, 95% CI 0.87–0.94) between these four treatment regimens.

**Conclusions:**

The meta-analysis indicates that DA-EPOCH could be more effective in providing curative treatment for adult Burkitt lymphoma patients, especially without CNS and BM involvement considering OS time. Due to the types of studies and the limited number of included studies, bias should be acknowledged and a randomized controlled trial (RCT) needs to be performed to further identify the optimal treatment regimen for such patients.

## Introduction

Burkitt lymphoma (BL) is a highly aggressive B-cell non-Hodgkin’s lymphoma, which often involves extranodal sites or is represented as leukemia (1). It is rare and characterized by rapid tumor cell proliferation, which one deemed related to EB virus infection and chromosomal translocation involving the c-myc oncogene (2, 3).

Current national guidelines recommend short-term, intensive, and multi-drug regimens as the first-line treatment option for adult BL patients (4), including CODOX-M/IVAC, DA-EPOCH, and Hyper-CVAD. GMALL-B-ALL/NHL2002 is also commonly used for the treatment of BL patients and has achieved a promising outcome in already published reports (5–7). Clinical trials and systematic reviews have demonstrated that the anti-CD20 monoclonal antibody rituximab in addition to chemotherapies further provides efficacy benefits in patients with BL in the advent of the immunotherapy era (8, 9). Rituximab has become a component of standard chemotherapy in the treatment of BL for years, and no comparative studies have been conducted to evaluate the efficacy of these first-line regimens so far. Here, we conducted this meta-analysis to identify the better regimen by accessing overall survival (OS), progression-free survival (PFS), and overall response rate (ORR) data in the literature up to December 2021.

## Methods

### Literature search strategy

The meta-analysis was performed in accordance with the Preferred Reporting Items for Systematic Review and Meta-Analysis (PRISMA) guidelines using four scientific databases, PubMed, Embase, Web of Science, and Cochrane Central Register of Controlled Trials (CENTRAL) Web of Science from 10 December 2021 to 5 May 2022 (10). The following search terms were used: (“Burkitt Lymphoma” [Mesh]) OR (((Burkitt lymphoma [Title/Abstract]) OR (Burkitt’s lymphoma [Title/Abstract])) OR (BL [Title/Abstract])) AND ((“Rituximab” [Mesh])) OR (Rituximab [Title/Abstract]). The detailed search terms are listed in **Table S1**.

### Eligibility criteria

Because the aim of this study was to assess different regimens, inclusion criteria were as follows: (1) prospective and retrospective studies; (2) English language articles and full texts; (3) chemotherapy regimens containing rituximab; and (4) studies that reported survival and response for at least 2 years. Exclusion criteria were as follows: (1) children (patient’s age ≤ 14 years old); (2) sample number of fewer than 10 patients; and (3) article type: conference abstract, letter, comment, and other types that reported incomplete information. The detailed excluded studies for full-text screening are listed in **Table S2**.

Included prospective non-randomized studies were assessed using the MINORS index (11). Included retrospective studies were used to evaluate the quality using the JBI Critical Appraisal Checklist (12).

### Data extraction

The following records about basic information of each study were extracted independently by two authors (XL and YL): study type, published journal, name of the first author, nations where trials were conducted, year of publication, chemotherapy regimen, the total number of patients, 2-year OS rate, 2-year PFS rate, ORR (CR and PR), and follow-up time. Patients’ characteristics including median age, gender, human immunodeficiency virus (HIV)-positive, LDH levels, the proportion of stage III–IV, International Prognostic Index (IPI) or Eastern Cooperative Oncology Group (ECOG) score >2, and the number of patients with central nervous system (CNS) and bone marrow (BM) involvement were also collected. The other authors (LQ and RL) resolved any inconsistencies in the data extraction process.

### Statistical analysis

The primary outcome for efficacy was a 2-year OS rate; the second outcomes were a 2-year PFS rate and ORR. We used the software GetData Graph Digitizer 2.26.0.20 to digitize and extract data when the above-mentioned outcomes were presented only as a Kaplan–Meier survival graph. Heterogeneity was assessed using the *I* ^2^ statistic. A forest plot was applied in the random-effects model if significant heterogeneity was observed (*I* ^2^ > 50%); the fixed model would be applied otherwise. All of them used the DerSimonian and Laird method. The normality test results were used to determine whether the proportions should be applied in untransformed data or transformed with the Freeman–Tukey double arcsine transformation and confidence intervals were calculated using the Jackson method. A meta-regression was also performed to examine factors possibly related to outcomes, including median age, gender, HIV-positive, the proportion of elevated LDH, high risk, stage III–IV, CNS, and BM involvement. Potential publication bias was assessed by inspecting a funnel plot and Egger’s test. All statistical tests were two-tailed, and *p* values < 0.05 were considered significant. R software environment version 4.1.2 was used for statistical computing and graphics.

## Results

### Literature search

**Figure 1:**
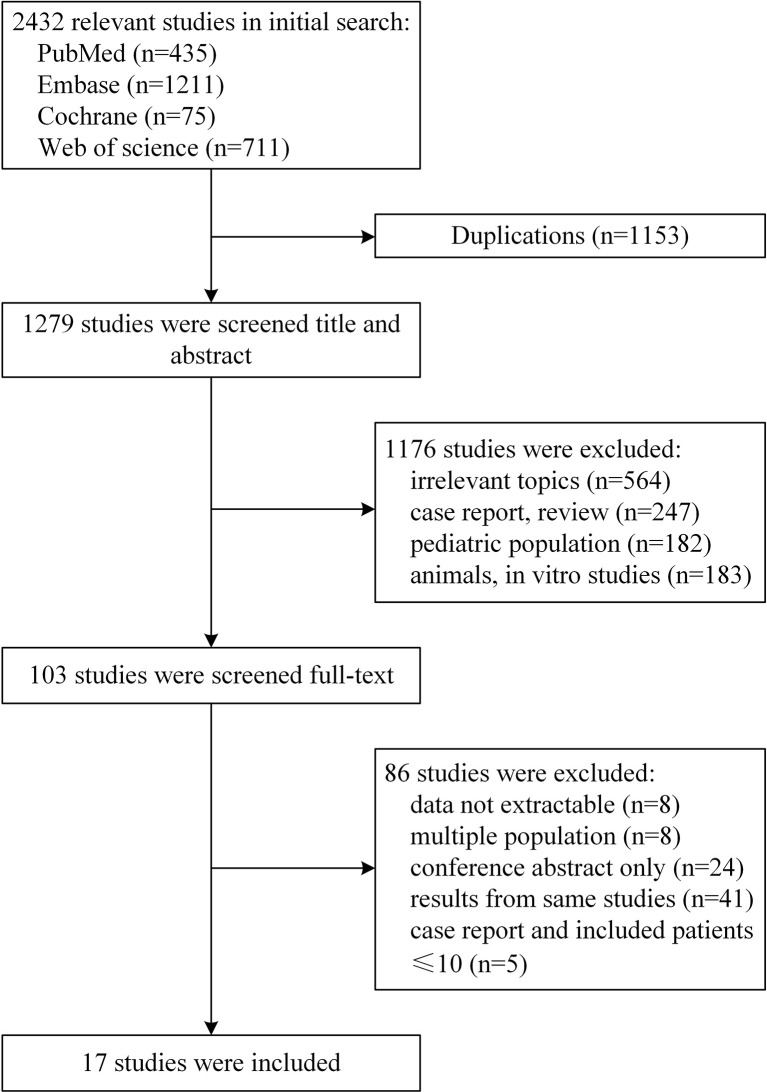
Flow diagram of the study selection process.

A total of 2,432 potentially relevant studies were identified for screening (**Figure 1**). After excluding 1,153 duplications, 103 studies remained by accessing titles and abstracts. Among the remaining full-text selections, 86 articles were removed because of conference posters, overlapping data, information being unable to extract, multiple cohorts, and small sample size. A total of 17 studies including altogether 1,258 patients were included in our final analysis (5–7, 13–26).

### Study characteristics

The baseline characteristics for the 17 studies are presented in **Table 1**. Twelve included articles were prospective studies, of which only one was a comparative study, and the remaining 11 were non-comparative and single-arm studies. In addition, six retrospective articles were included. No randomized controlled trial (RCT) was included. All of the articles were published between 2006 and 2021, comprising 1,258 patients. A total of 1,258 patients were included, with the number of patients in each study ranging from 14 to 363. The age range was 15 to 88 years (5, 19), with median ages between 25 and 52 years (19, 24). A relatively higher proportion of male patients was observed in the range from 0.6 to 0.889, which was consistent with the results of previous studies (27, 28). The included patients were followed up for 1.4 to 147.6 months. Five cohorts included only HIV-negative patients, three cohorts included only HIV-positive patients, and the proportion of HIV-positive patients in the remaining groups was 0.14 to 0.323. Four treatment groups were as follows: eight cohorts from eight studies with CODOX-M/IVAC (13–15, 17, 21, 22, 24, 25), three cohorts from two studies with DA-EPOCH (19, 20), three cohorts from three studies with GMALL-B-ALL/NHL2002 (5–7), and four cohorts from four studies with Hyper-CVAD, all of which used rituximab (16, 18, 23, 26). The included studies had a median follow-up time of 35 months (range, 20 to 86) (19, 23).

**Table 1:** Baseline characteristics of included studies.

First author	Publication year	No. of patients	Group	Age	Male %	Follow-up	Study type
Mohamedbhai	2010	14	CODOX-M/IVAC	NA (22–65)	0.857	34	Retrospective
Corazzelli	2011	30	CODOX-M/IVAC	52 (25–77)	0.7	36	Prospective
Evens	2013	25	CODOX-M/IVAC	44 (23–70)	0.88	34	Prospective
Alwan	2015	42	CODOX-M/IVAC	46 (25–69)	0.786	21	Retrospective
Noy	2015	34	CODOX-M/IVAC	42 (19–55)	0.882	26	Prospective
Zhu	2018	81	CODOX-M/IVAC	47 (18–72)	0.79	56.4	Prospective
Phillips	2020	27	CODOX-M/IVAC	35 (20–64)	0.889	56.9	Prospective
Chen	2021	123	CODOX-M/IVAC	36 (18–69)	0.65	43.2	Retrospective
Dunleavy	2013	19	DA-EPOCH	25 (15–88)	0.68	86	Prospective
Dunleavy	2013	11	DA-EPOCH	44 (24–60)	0.82	73	Prospective
Roschewski	2020	113	DA-EPOCH	49 (18–86)	0.79	58.7	Prospective
Ribera	2013	118	GMALL-B-ALL/NHL2002	44 (15–83)	0.72	30	Prospective
Intermesoli	2013	105	GMALL-B-ALL/NHL2002	47 (17–78)	0.6	23.8	Prospective
Hoelzer	2014	363	GMALL-B-ALL/NHL2002	42 (16–85)	0.7	43.2	Prospective
Thomas	2006	31	Hyper-CVAD	46 (17–77)	0.77	22	Prospective
Hong	2015	43	Hyper-CVAD	51 (20–83)	0.674	20	Retrospective
Malkan	2016	25	Hyper-CVAD	39 (16–63)	0.72	22.7	Retrospective
Samra	2021	54	Hyper-CVAD	42 (18–77)	0.67	50	Retrospective

### Study quality assessment

The summary of the quality assessment is outlined in **Table 2**. One study including two comparative groups scored 21 on the MINORS index (19). Ten prospective single-arm studies had generally moderate quality scoring between 13 and 15 points (5–7, 14–17, 19, 20, 24, 25). Six retrospective studies were determined to include after quality evaluation by the JBI Critical Appraisal Checklist (13, 18, 21–23, 26).

**Table 2:** Quality assessment of included studies.

MINORS index for included non-randomized studies.													
Study	Q1	Q2	Q3	Q4	Q5	Q6	Q7	Q8	Q9	Q10	Q11	Q12	Total
Phillips 2020	2	2	2	2	0	2	2	2					14
Noy 2015	2	2	2	2	0	2	2	2					14
Thomas 2006	2	2	2	2	0	2	2	1					13
Ribera 2013	2	2	2	2	1	2	2	1					14
Evens 2013	2	2	2	2	0	2	2	1					13
Intermesoli 2013	2	2	2	2	0	2	2	1					13
Hoelzer 2014	2	2	2	2	1	2	2	2					15
Roschewski 2020	2	2	2	2	0	2	2	1					13
Corazzelli 2011	2	2	2	2	0	2	2	1					13
Zhu 2018	2	2	2	2	0	2	2	1					13
Dunleavy 2013	2	2	2	2	0	2	2	1	2	2	2	2	21

JBI Critical appraisal checklist for case series for included retrospective studies.												
Study	Q1	Q2	Q3	Q4	Q5	Q6	Q7	Q8	Q9	Q10	Q11	Overall appraisal
Alwan 2015	yes	yes	Yes	Yes	Yes	Yes	Yes	Yes	Yes	Yes	Yes	Include
Samra 2021	NA	NA	Yes	Yes	Yes	Yes	Yes	Yes	Yes	Yes	Yes	Include
Chen 2021	NA	NA	Yes	Yes	Yes	Yes	Yes	Yes	Yes	Yes	Yes	Include
Sajir 2010	NA	NA	Yes	Yes	No	No	Yes	Yes	Yes	Yes	Yes	Include
Hong 2015	NA	NA	Unclear	NA	Yes	Yes	Yes	Yes	Yes	Yes	Yes	Include
Malkan 2016	yes	yes	Yes	Yes	Yes	Yes	Yes	Yes	Yes	Yes	Yes	Include

*NA, Not Applicable.

### Pooled analyses and meta-regression

#### Primary outcome

**Figure 2:**
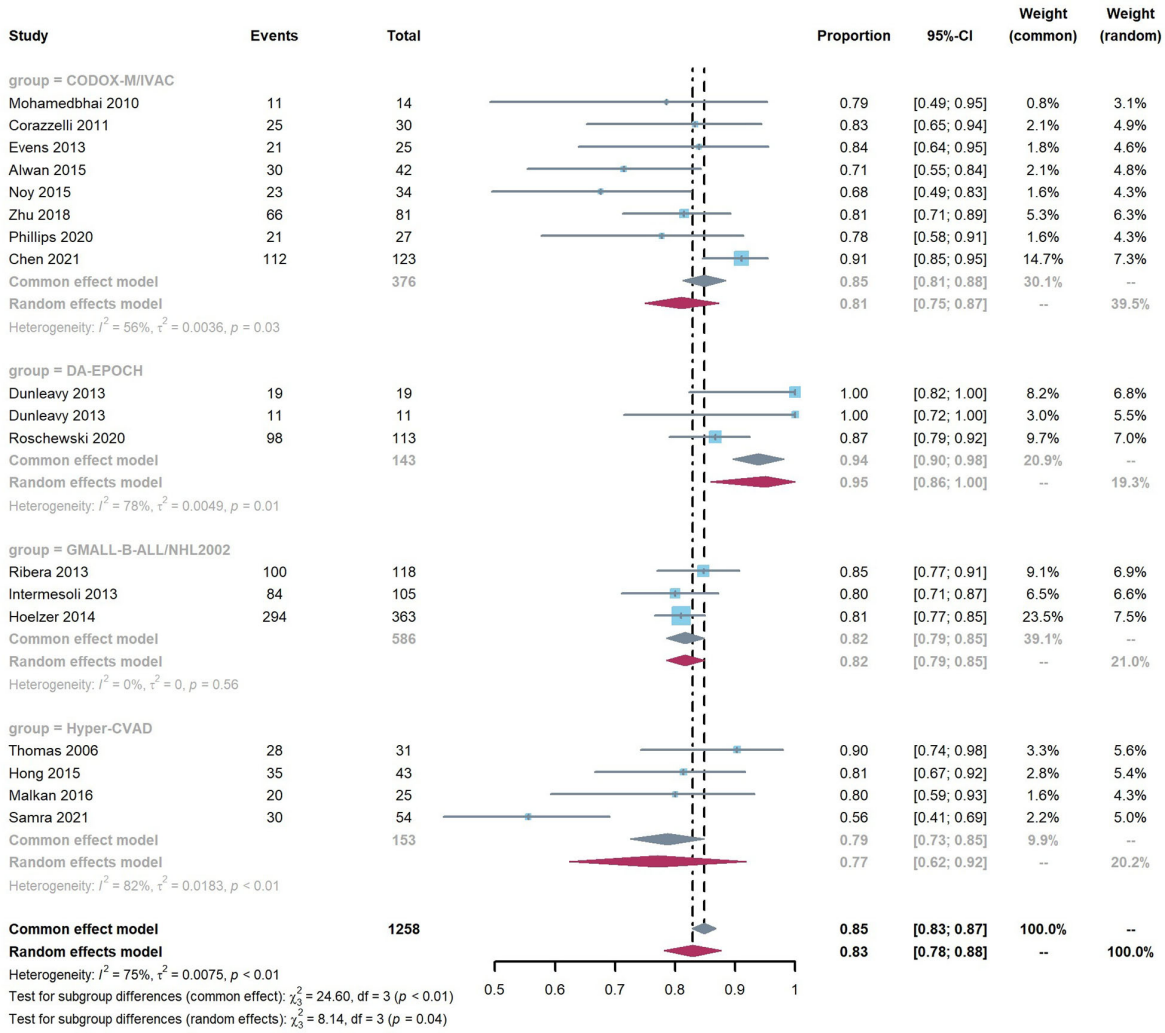
Pooled 2-year OS rate according to regimen group.

**Figure 3:**
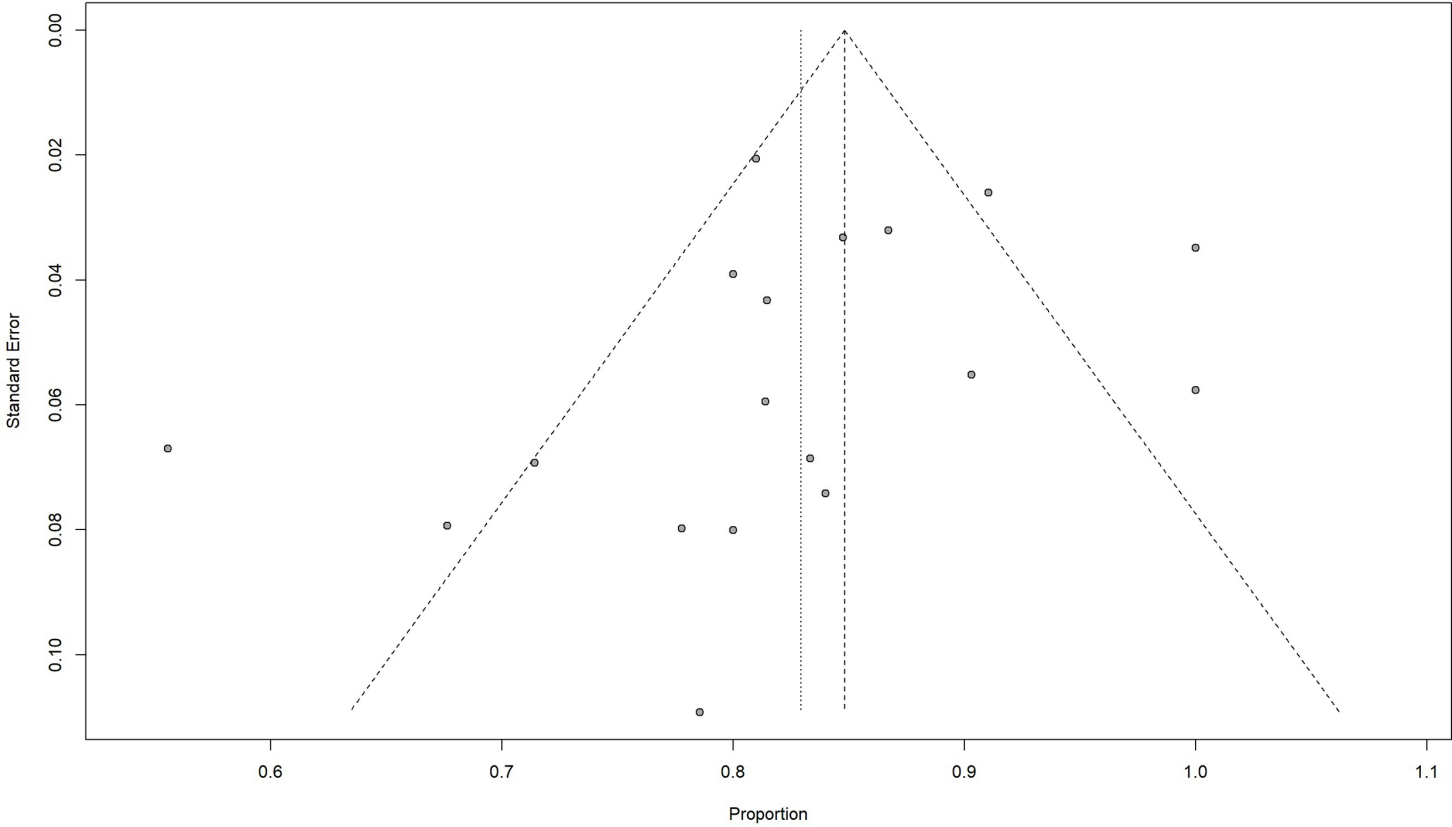
Funnel plot of 2-year OS rate.

The overall pooled 2-year OS rate was 0.83 (95% CI 0.78–0.88). Our result showed a significant difference in the 2-year OS rate according to the chemotherapy regimen groups (*p* < 0.05). The results favored the DA-EPOCH group, which led to a higher OS rate (0.95, 95% CI 0.86–1.00), compared to CODOX-M/IVAC (0.81, 95% CI 0.75–0.87), Hyper-CVAD (0.77, 95% CI 0.62–0.92), and GMALL-B-ALL/NHL2002 (0.82, 95% CI 0.79–0.85) (**Figure 2**). Meta-regression with a mixed-effects model demonstrated that elevated LDH, the proportion of stage III-IV and high-risk patients, and the involvement of CNS or BM are associated with significant heterogeneity (*I* ^2^ = 75%, *p* < 0.01) (**Table 3**). By contrast, no difference was found in age, sex, and HIV infection among different regimen groups. The funnel plot was considered roughly symmetric by inspection (**Figure 3**). Egger’s test was also performed to detect publication bias for included studies (*p* = 0.2519).

**Table 3:** Meta‐regression analysis in relation to 2‐year OS rate.

Variables	Coefficient	Standard error	95% CI	*p*
Median age (years)	−0.0051	0.0031	−0.0111 to 0.001	0.0991
Male gender (%)	−0.0814	0.2773	−0.6249 to 0.4621	0.7691
HIV positive (%)	−0.0423	0.076	−0.1913 to 0.1068	0.5783
Elevated LDH (%)	−0.2472	0.1089	−0.4605 to −0.0338	0.0232
Stage III–IV (%)	−0.4481	0.1542	−0.7504 to −0.1459	0.0037
HR (%)	−0.153	0.0584	−0.2676 to −0.0385	0.0088
CNS involvement (%)	−0.6144	0.2084	−1.0229 to −0.206	0.0032
BM involvement (%)	−0.325	0.082	−0.4858 to −0.1642	<.0001

#### Secondary outcomes

The pooled ORR was 0.90 (95% CI 0.87–0.94) with significant heterogeneity (*I* ^2^ = 69%, *p* < 0.01) in connection with the elevated LDH. There was no significant difference (*p* = 0.07) in ORR between DA-EPOCH, CODOX-M/IVAC, Hyper-CVAD, and GMALL-B-ALL/NHL2002 groups, with values of 0.99 (95% CI 0.93–1.00), 0.92 (95% CI 0.86–0.97), 0.89 (95% CI 0.79–0.99), and 0.86 (95% CI 0.79–0.94), respectively (**Figure S1**). A similar result was found in that these four groups DA-EPOCH (0.92, 95% CI 0.82–1.00), CODOX-M/IVAC (0.80, 95% CI 0.76–0.85), Hyper-CVAD (0.71, 95% CI 0.57–0.86), and GMALL-B-ALL/NHL2002 (0.79, 95% CI 0.76–0.83) have no obvious difference in the 2-year PFS rate (**Figure S2**). The roughly same factors as ORR were identified in the heterogeneity analysis of PFS (*I* ^2^ = 61%, *p* < 0.01) using meta-regression, except for LDH. No publication bias was observed in both ORR and 2-year PFS rates using the funnel plot and Egger’s test (**Figures S3**, **S4**).

## Discussion

BL is an aggressive and chemotherapy-sensitive B-cell non-Hodgkin’s lymphoma categorized into endemic, sporadic, and immunodeficiency-associated subtypes (29). The majority of BL patients obtained long-term survival after some intensive chemotherapy, and the prognosis has further improved owing to the advent of rituximab. Research showed that rituximab, the anti-CD20 monoclonal antibody, can not only prolong the time of disease progression but also extend OS for a variety of B-cell lymphomas, including BL (30). It is still uncertain which is the optimal regimen containing rituximab for BL patients, and we aim to answer this question through a meta-analysis. Previous studies found heterogeneity between pediatric and adult BL patients; thus, this meta-analysis focused on patients no less than 14 years of age (28). A total of 17 studies and 1,258 patients were finally identified through data search and literature screening and divided into four treatment groups as follows: DA-EPOCH, CODOX-M/IVAC, Hyper-CVAD, and GMALL-B-ALL/NHL2002. All included patients were sporadic or immunodeficiency-associated types, probably because endemic BL is prevalent in children of sub-Saharan Africa (29).

CODOX-M/IVAC, developed by Magrath, is a highly effective alternate and the most commonly used regimen as our result showed that patients in almost half of the included studies were treated with this chemotherapy program (31). B-NHL2002, a short intensive chemotherapy program based on a pediatric Berlin-Frankfurt-Münster protocol, was updated and improved several times by the German Multicenter Study Group for Adult ALL (GMALL). Three current reported large prospective trials showed substantial cure rates in adult BL/leukemia whether HIV-negative or HIV-positive (5–7). Hyper-CVAD (hyper-fractionated cyclophosphamide, doxorubicin, vincristine, and dexamethasone alternating with methotrexate plus cytarabine) was also a comparable treatment option among established dose-intensive regimens for BL (18, 23, 26). A retrospective study by Samra et al. reported that the Hyper-CVAD protocol showed highly promising efficacy and safety in high-risk patients with CNS or BM involvement (18). The DA-EPOCH (Risk-adapted etoposide, prednisone, vincristine, cyclophosphamide, and doxorubicin) protocol has been widely used in lymphoma and is well tolerated by BL patients of all ages and those with HIV infection (20). Based on the results of our meta-analysis, the DA-EPOCH regimen group might be considered a better treatment option for adult BL patients by comparing the 2-year OS rate among the four regimen groups. No significant difference was observed in the 2-year PFS rate and ORR among these four groups. Interestingly, one RCT performed by Chamuleau reported a similar estimated 2-year OS among R-CODOX-M/R-IVAC and DA-EPOCH-R for high-risk BL; this trial was suspended because of the slow accrual rate. The limited sample size and inclusion of only high-risk populations in this trial may have contributed to the discrepant results between this RCT and our analysis (32).

LDH levels, stage III–IV, CNS, and BM involvement have been widely used for risk stratification and considered as independent influential factors in the poor prognosis of BL. Our result displayed various sources of heterogeneity, including LDH levels, stage III–IV, CNS, and BM involvement, consistent with the previously reported results in strong association with OS rate, further confirming the reliability of our meta-analysis (33–35). Previous studies showed that BL is highly aggressive, especially when with extensive involvement. Patients with BM involvement using the Ann Arbor staging system were diagnosed at stage IV, and those with CNS involvement had no detailed description. The St Jude/Murphy system, which was much more commonly used for staging, suggested that BL patients with BM and/or CNS involvement were divided into stage IV and the high-risk group. Anyhow, the involvement of CNS and/or BM directly related to the advanced stage was a significant factor associated with inferior prognosis. As shown in our results, because of the different proportions of CNS and/or BM involvement in the included studies and the identification of the source of heterogeneity between patients with and without CNS and BM involvement by meta-regression, the conclusion that “the DA-EPOCH regimen had a higher OS rate compared with three other treatment groups” seems to be inapplicable to patients with BM or CNS involvement. Furthermore, studies have shown no difference in clinical and biochemical characteristics and treatment outcomes between HIV-positive and HIV-negative cohorts after rituximab-based chemotherapy (35–38). We agreed that HIV status does not affect the prognosis of adult BL patients. The prevalence of BL is higher in men than in women; however, we found no gender differences regarding prognosis.

In this meta-analysis, we compared the efficacy of different chemotherapy regimens by subgroup analysis and identified the factors that may influence the treatment outcomes. This is the first answer to the question of which is the best treatment option for BL in the rituximab era. The DA-EPOCH-R regimen was a less toxic regimen than other dose-intensive regimens for BL (e.g., CODOX-M-IVAC) (39). DA-EPOCH-R was a preferred option not only because it extends survival time but also because of its better safety and lower medical costs.

The following limitations of this study should be acknowledged. Firstly, included articles were performed by different study types, and most of the included studies were single-arm without RCT studies. Secondly, the small number of included studies and patients may not be representative enough of all adult BL patients; there were only less than 20 patients in some included studies. Thirdly, doses of chemotherapy agents modified by medicine centers and various manufacturers of rituximab cannot be overlooked. Also, the results of our heterogeneity analysis could be different from the real world owing to unavailable data from some cohorts. Furthermore, the applicability of our results in patients with BM and/or CNS involvement remains to be further validated due to the existence of heterogeneity.

## Conclusion

In summary, a better treatment strategy for prolonging the survival time of adult BL without CNS and/or BM involvement was first identified through our meta-analysis. We found that DA-EPOCH had a greater OS rate in contrast with CODOX-M/IVAC, GMALL-B-ALL/NHL2002, and Hyper-CVAD groups. More studies, especially RCTs, need to be performed to identify the optimal chemotherapy regimen for patients with extensive organ involvement.

## Data availability statement

The original contributions presented in the study are included in the article/**Supplementary Material**. Further inquiries can be directed to the corresponding author.

## Author contributions

XL: Data acquisition, writing the original draft, and editing. YL: Data acquisition and analysis. RL: Resources, and data acquisition. JL: Manuscript revision and project administration. XY: Providing guidance, manuscript revision, and funding acquisition. LQ: Methodology, manuscript revision, funding acquisition and final approval of the last version. All authors contributed to the article and approved the submitted version.

## Supplementary material

Supplementary Figure 1 Pooled ORR according to regimen group

Supplementary Figure 2 Pooled 2-year PFS rate according to regimen group

Supplementary Figure 3 Funnel plot of ORR

Supplementary Figure 4 Funnel plot of 2-year PFS rate
