# Supplementary material for: Comparison of chemotherapy regimens plus rituximab in adult Burkitt lymphoma: A single-arm meta-analysis
Source: Front Oncol. 2022 Dec 23;12:1063689. doi: 10.3389/fonc.2022.1063689 (PMC9816660; doi:10.3389/fonc.2022.1063689)
Supplement: Supplementary file 1 [file Table_1.docx]

Supplementary Table S1 Detailed search strategy

| Medline (via PUBMED) | |
| --- | --- |
| Population | ("Burkitt Lymphoma"[Mesh]) OR (((Burkitt lymphoma [Title/Abstract]) OR (Burkitt's lymphoma[Title/Abstract])) OR (BL[Title/Abstract])) |
| Intervention | **(("Rituximab"[Mesh])) OR (rituximab[Title/Abstract])** |
| Comparision | Omitted |
| Outcome | Omitted |
| Study design | Omitted |
| ECR filter | ("Burkitt Lymphoma"[Mesh]) OR (((Burkitt lymphoma [Title/Abstract]) OR (Burkitt's lymphoma[Title/Abstract])) OR (BL[Title/Abstract])) AND **(("Rituximab"[Mesh])) OR (rituximab[Title/Abstract])** |
| Number | 435 |
| Embase | |
| Population | 'burkitt lymphoma'/exp OR 'burkitt lymphoma' |
| Intervention | 'Rituximab'/exp OR ' Rituximab' |
| Comparision | Omitted |
| Outcome | Omitted |
| Study design | Omitted |
| ECR filter | ('burkitt lymphoma'/exp OR 'burkitt lymphoma') AND ('Rituximab'/exp OR ' Rituximab') AND ([adolescent]/lim OR [adult]/lim OR [aged]/lim OR [middle aged]/lim OR [very elderly]/lim OR [young adult]/lim) |
| Number | 1211 |
| Cochrane Central Register of Controlled Trials (CENTRAL) | |
| Population | MeSH descriptor: [Burkitt Lymphoma] explode all trees OR (“Burkitt”):ti,ab,kw |
| Intervention | MeSH descriptor: [rituximab] explode all trees OR (“rituximab”):ti,ab,kw |
| Comparision | Omitted |
| Outcome | Omitted |
| Study design | Omitted |
| ECR filter | [Burkitt Lymphoma] explode all trees OR (“Burkitt”) AND [rituximab] explode all trees OR (“rituximab”) |
| Number | 75 |
| Web of science | |
| Population | TS=(Burkitt Lymphoma OR Burkitt's Lymphoma OR BL) |
| Intervention | **TS=(rituximab)** |
| Comparision | Omitted |
| Outcome | Omitted |
| Study design | Omitted |
| ECR filter | TS=(Burkitt Lymphoma OR Burkitt's Lymphoma OR BL) AND **(rituximab)** |
| Number | 711 |
