# Supplementary material for: Comparison of chemotherapy regimens plus rituximab in adult Burkitt lymphoma: A single-arm meta-analysis
Source: Front Oncol. 2022 Dec 23;12:1063689. doi: 10.3389/fonc.2022.1063689 (PMC9816660; doi:10.3389/fonc.2022.1063689)
Supplement: Supplementary file 2 [file Table_2.docx]

Supplementary Table S2 Reasons of exclusion for studies in the full text assessment

| Study | Reason for exclusion |
| --- | --- |
| Ribera JM. Specific Intensive Chemotherapy Plus Rituximab for Advanced Burkitt's Lymphoma or Leukemia in Hiv-Positive and Negative Adult Patients. Blood (2007). | conference abstract only |
| Rizzieri DA. Efficacy and Toxicity of Rituximab and Brief Duration, High Intensity Chemotherapy with Filgrastim Support for Burkitt or Burkitt - Like Leukemia/Lymphoma: Cancer and Leukemia Group B (Calgb) Study 10002. Blood (2010). | conference abstract only |
| Bonifacio M. Short-Term, Intensive Chemotherapy Regimen Is Equally Effective in Burkitt Lymphoma (Bl) and in the Novel Who 2008 Entity “B-Cell Lymphoma, Intermediate between Dlbcl and Bl”. Haematologica (2011). | conference abstract only |
| Frairia C. Rituximab Plus Dose Intense Rapid-Cycling Chemotherapy with Intrathecal Cns Prophylaxis in Patients with Burkitt Lymphoma (Bl) and Intermediate Unclassifiable Diffuse Large B-Cell Lymphoma/Bl: Single Institution Experience. Blood (2011). | conference abstract only |
| Frairia C. Dose Intense Rapid-Cycling Chemotherapy Associated with Rituximab and Intrathecal Cns Prophylaxis in Patients with Burkitt Lymphoma (Bl) and Intermediate Unclassifiable Diffuse Large B Cell Lymphoma/Bl: Report of a Single Institution Experience. Haematologica (2011). | conference abstract only |
| Intermesoli T. Cure Rates and Toxicity Vary According to Age < Vs. > 55 Years in B-All and Burkitt Lymphoma Treated with the German Chemotherapy Plus Rituximab Protocol: Italian Study on over 100 Patients. Haematologica (2011). | conference abstract only |
| Oosten L. Treatment of Burkitt Lymphoma in Adults Using an Adapted Pediatric All Protocol: A Single Center Experience. Haematologica (2011). | conference abstract only |
| Pohlen M. Efficacy and Toxicity of a Rituximab and Methotrexate Based Regimen (Gmall B-All/Nhl 2002 Protocol) in Burkitt's and Primary Mediastinal Large B-Cell Lymphoma. American journal of hematology (2011). | conference abstract only |
| Pohlen MY. Primary Mediastinal Large B-Cell and Burkitt's/Burkitt-Like Lymphoma: Efficacy and Toxicity of a Rituximab and Methotrexate Based Regimen (Gmall B-All/Nhl 2002 Protocol). Onkologie (2011). | conference abstract only |
| Wildes TM. Hyper-Cvad and Rituximab-Hypercvad in Burkitt Lymphoma (Bl): A Multi-Institutional Experience. Journal of Clinical Oncology (2011). | conference abstract only |
| Mee M. Improved Outcomes in Hiv-Associated Burkitt's Lymphoma with Codox-M +/- Ivac Combined with Rituximab and Cart. HIV medicine (2013). | conference abstract only |
| Prica A. Rituximab Improves Overall Survival in Patients Treated with Codox-M/Ivac for Burkitt Lymphoma (Bl) and B-Cell Lymphoma, Unclassifiable, with Features Intermediate between Diffuse Large B-Cell Lymphoma and Bl: A Single Center Experience and Review of the Literature. Blood (2013). | conference abstract only |
| Mariotti J. High Cure Rates of the Short-Term Gitil Chemotherapy Programme for Adult Patients with Burkitt Lymphoma. Blood (2014). | conference abstract only |
| Shahbazi S. Prolonged Low Intensity Epoch-Rituximab Has Improved Toxicity in Burkitt Lymphoma Compared with Standard Short, High Intensity Therapy. Cancer biology & therapy (2014). | conference abstract only |
| Regragui S. Toxicity and Efficacy of Lmba-02 Protocol in the Treatment of Burkitt's Lymphoma among the Moroccan Adult Population. Haematologica (2015). | conference abstract only |
| Zhu KY. Population-Based Survival Outcomes in Adult Patients with Burkitt Lymphoma (Bl) Treated with Cyclophosphamide, Vincristine, Doxorubicin, High-Dose Methotrexate (Codoxm)/ Ifosfamide, Etoposide and High-Dose Cytarabine (Ivac) Plus or Minus Rituximab (R) in British Columbia (Bc), Canada. Blood (2016). | conference abstract only |
| de Olano VC. Leukemia/Lymphoma of Burkitt in the Era of Rituximab. 10 Years of Experience of a Single Center. Haematologica (2017). | conference abstract only |
| Von Wolff M. Treatment of Burkitt/Burkitt-Like Lymphoma - Impact of Autologous Transplantation in First Line Therapy. Oncology research and treatment (2017). | conference abstract only |
| Marangon M. The Berlin-Frankfurt-Münster Protocol for the Upfront Treatment of Aggressive Lymphomas: The Bologna Experience. American journal of hematology (2018). | conference abstract only |
| Ahmed A. Outcome of 62 Patients with Burkitt Lymphoma: Local Experience from Princess Noorah Oncology Center – King Abdulaziz Medical City – Jeddah – Saudi Arabia from 2000 to 2017. Clinical Lymphoma, Myeloma and Leukemia (2019). | conference abstract only |
| Bettencourt Medeiros P. Safety and Efficacy of Dose-Intensive Chemotherapy Including Rituximab in the Treatment of Burkitt's Lymphoma/Leukemia Is Independent of Hiv Status. HemaSphere (2020). | conference abstract only |
| Boltezar L. Survival of Adult Burkitt Lymphoma Patients Treated with the Nhl-Bfm 90 Protocol in a 10-Year Period in Slovenia. Annals of Oncology (2020). | conference abstract only |
| Decker DP. Treatment Strategies and Risk of Central Nervous System Recurrence in High-Grade B-Cell and Burkitt Lymphoma. Leukemia & lymphoma (2020). | conference abstract only |
| Samra B. All-326: Low Incidence of Central Nervous System (Cns) Relapse with Hyper-Cvad-R Regimen in Adults with Burkitt Lymphoma/Leukemia (Bl) and High-Grade B-Cell Lymphoma (Hgbcl). Clinical Lymphoma, Myeloma and Leukemia (2020). | conference abstract only |
| Thomas DA. Rituximab and Hyper-Cvad for Adult Burkitt's (Bl) or Burkitt's-Like (Bll) Leukemia or Lymphoma. Blood (2001). | results from same studies |
| Thomas DA. Outcome with the Hyper-Cvad and Rituximab Regimen in Burkitt (Bl) and Burkitt-Like (Bll) Leukemia/Lymphoma. Blood (2004). | results from same studies |
| Nct. Lmba02 Protocol for Patients with a Burkitt Lymphoma. https://clinicaltrialsgov/show/NCT00180882 (2005). | results from same studies |
| Thomas DA. Hyper-Cvad and Rituximab Therapy in Hiv-Negative Burkitt (Bl) or Burkitt-Like (Bll) Leukemia/Lymphoma and Mature B-Cell Acute Lymphocytic Leukemia (B-All). Journal of Clinical Oncology (2005). | results from same studies |
| Nct. Doxorubicin Hydrochloride Liposome and Rituximab with Combination Chemotherapy in Treating Patients with Newly Diagnosed Burkitt's Lymphoma or Burkitt-Like Lymphoma. https://clinicaltrialsgov/ show/NCT00392990 (2006). | results from same studies |
| Dunleavy K. Efficacy and Toxicity of Dose-Adjusted Epoch-Rituximab in Adults with Newly Diagnosed Burkitt Lymphoma. Journal of Clinical Oncology (2007). | results from same studies |
| Fayad L. Update of the M. D. Anderson Cancer Center Experience with Hyper-Cvad and Rituximab for the Treatment of Mantle Cell and Burkitt-Type Lymphomas. Clinical lymphoma & myeloma (2007). | results from same studies |
| Abramson JS. Rituximab Added to Codox-M/Ivac Is Highly Effective in Hiv-Negative and Hiv-Positive Burkitt Lymphoma. Blood (2008). | results from same studies |
| Dunleavy K. A Prospective Study of Dose-Adjusted (Da) Epoch with Rituximab in Adults with Newly Diagnosed Burkitt Lymphoma: A Regimen with High Efficacy and Low Toxicity. Annals of Oncology (2008). | results from same studies |
| Oriol A. High-Dose Chemotherapy and Immunotherapy in Adult Burkitt Lymphoma: Comparison of Results in Human Immunodeficiency Virus-Infected and Noninfected Patients. Cancer (2008). | results from same studies |
| Thomas DA. Long-Term Outcome after Hyper-Cvad and Rituximab Chemoimmunotherapy for Burkitt (Bl) or Burkitt-Like (Bll) Leukemia/Lymphoma and Mature B-Cell Acute Lymphocytic Leukemia (All). Blood (2008). | results from same studies |
| Barnes BA. Rituximab Added to Codox-M/Ivac Has No Clear Benefit Compared to Codox-M/Ivac Alone in Adult Patients with Burkitt Lymphoma. Blood (2009). | results from same studies |
| Dunleavy K. Dose-Adjusted Epoch with Rituximab (Da-Epoch-R) Has High Efficacy and Low Toxicity in Young Adults with Newly Diagnosed Burkitt Lymphoma: A Prospective Study of 25 Patients. Haematologica Meeting Reports (2009). | results from same studies |
| Mohamedbhai SG. Rituximab in Combination with Codox-M/Ivac: Toxicity and Efficacy in 17 Adults with Non-Hiv Related B-Cell Non-Hodgkin Lymphomas with >95% Proliferation Index. British journal of haematology (2009). | results from same studies |
| Dujmovic D. Addition of Rituximab to High-Dose Methotrexate-Based Chemotherapy Improves Outcomes in Adult Burkitt Lymphoma Patients. Haematologica (2010). | results from same studies |
| Gregory SA. Incorporation of Rituximab and Liposomal Doxorubicin into Codox-M/Ivac for Hiv-Negative and Hiv-Positive Adult Patients (Pts) with Untreated Burkitt's Lymphoma (Bl): Preliminary Results of a Multicenter Phase Ii Study. Journal of Clinical Oncology (2010). | results from same studies |
| Nct. Phase Ii Study of Dose-Adjusted Epoch-Rituximab in Adults with Untreated Burkitt Lymphoma and C-Myc+ Diffuse Large B-Cell Lymphoma. https://clinicaltrialsgov/show/NCT01092182 (2010). | results from same studies |
| Ribera JM. Specific Chemotherapy and Rituximab in Hiv-Infected Patients with Burkitt's Leukemia or Lymphoma. Results of a German-Spanish Study and Analysis of Prognostic Factors. Blood (2010). | results from same studies |
| Thomas DA. Long-Term Outcome for De Novo or Minimally Treated Burkitt-Type Lymphoma/Leukemia (Bl/B-All) after Therapy with Hyper-Cvad and Rituximab. Blood (2010). | results from same studies |
| Evens AM. Integration of Rituximab and Liposomal Doxorubicin (Doxil (R)) into Codox-M/Ivac for Hiv-Negative and Hiv+ Adults with Untreated Burkitt's Lymphoma (Bl): Results of a Prospective Multicenter Phase Ii Study. Blood (2011). | results from same studies |
| Thomas DA. Hyper-Cvad and Rituximab for De Novo Burkitt Lymphoma/Leukemia. Blood (2011). | results from same studies |
| Ventre MB. Safety and Activity of Intensive Short-Term Chemoimmunotherapy in Hiv-Positive (Hiv+) Patients (Pts) with Burkitt Lymphoma (Bl). Blood (2011). | results from same studies |
| Evens AM. The Incorporation of Rituximab (R) and Liposomal Doxorubicin (Ld) into Codox-M/Ivac for Untreated Burkitt Lymphoma (Bl): Final Results of a Prospective Multicenter Phase Ii Study. Journal of Clinical Oncology (2012). | results from same studies |
| Hoelzer D. Substantially Improved Outcome of Adult Burkitt Non-Hodgkin Lymphoma and Leukemia Patients with Rituximab and a Short-Intensive Chemotherapy; Report of a Large Prospective Multicenter Trial. Blood (2012). | results from same studies |
| Intermesoli T. High Cure Rates in Burkitt Leukemia and Lymphoma: Nilg Study of the German Short Intensive Rituximab-Chemotherapy Program. Blood (2012). | results from same studies |
| Rodrigo JA. Hiv-Associated Burkitt Lymphoma: Good Efficacy and Tolerance of Intensive Chemotherapy Including Codox-M/Ivac with or without Rituximab in the Haart Era. Advances in hematology (2012). | results from same studies |
| Evens AM. Results of a Phase 2 Multicentre Study of the Addition of Rituximab to Codox-M/Ivac for Untreated Burkitts's Lymphoma: Impact of Plasma and Cerebrospinal Fluid Rituximub Levels. Hematological oncology (2013). | results from same studies |
| Intermesoli T. High Cure Rates in Burkitt Leukemia and Lymphoma: Nilg Study of the German Short Intensive Rituximab-Chemotherapy Program. Haematologica (2013). | results from same studies |
| Evens AM. Corrections to "a Multicenter Phase Ii Study Incorporating High-Dose Rituximab and Liposomal Doxorubicin into the Codox-M/Ivac Regimen for Untreated Burkitt's Lymphoma". Annals of oncology : official journal of the European Society for Medical Oncology (2014). | results from same studies |
| Evens AM. A Multicenter Phase Ii Study Incorporating High-Dose Rituximab and Liposomal Doxorubicin into the Codox-M/Ivac Regimen for Untreated Burkitt's Lymphoma (Vol 24, Pg 3076, 2013). Annals of Oncology (2014). | results from same studies |
| Xicoy B. Dose-Intensive Chemotherapy Including Rituximab Is Highly Effective but Toxic in Human Immunodeficiency Virus-Infected Patients with Burkitt Lymphoma/Leukemia: Parallel Study of 81 Patients. Leukemia & lymphoma (2014). | results from same studies |
| McMillan A. The Addition of Rituximab to Codox-M & Ivac in First Line Therapy of Poor Risk Burkitt Lymphoma (Ipi 3-5) Yields an Excellent Outcome: A Phase 2 Uk Ncri/Bloodwise Trial (Llr 04058). British journal of haematology (2016). | results from same studies |
| Dunleavy K. Risk-Adapted Therapy in Adults with Burkitt Lymphoma: Updated Results of a Multicenter Prospective Phase Ii Study of Da-Epoch-R. Hematological oncology (2017). | results from same studies |
| Ferreri AJM. Safety and Activity of a Dose-Dense Short-Term Chemoimmunotherapy in Hiv-Positive Patients with Burkitt Lymphoma (Hiv-Bl Pts): Final Results of the Carmen Phase Ii Trial. Blood (2017). | results from same studies |
| Roschewski M. Risk-Adapted Therapy in Adults with Burkitt Lymphoma: Results of Nci 9177, a Multicenter Prospective Phase Ii Study of Da-Epoch-R. Blood (2017). | results from same studies |
| Phillips E. High Discrepancy Rates in the Diagnosis of Burkitt Lymphoma: Pathology and Updated Survival Results from the Uk Ncri/Llr R-Codox-M Trial. British journal of haematology (2018). | results from same studies |
| Phillips EH. Favourable Outcomes with R-Codox-M/R-Ivac across All Subgroups of Aggressive High Grade B-Cell Lymphoma: Pathology and Updated Survival Results from a Phase 2 Uk Ncri/Llr Trial. HemaSphere (2018). | results from same studies |
| Ribera JM. Rituximab and Specific Therapy for Patients with Burkitt's Leukemia and Lymphoma. Results of the Burkimab14 Trial from the Spanish Pethema and Geltamo Groups in 80 Patients. Blood (2019). | results from same studies |
| Ferreri AJM. Safety and Efficacy of the “Carmen” Regimen, a New Dose-Dense Short-Term Therapy in Patients with Aggressive B-Cell Lymphoma and Myc Rearrangement. Hematological oncology (2021). | results from same studies |
| Lakhotia R. Prognostic Factors Other Than Age Drive the Risk of Disease Progression in Adults with Burkitt Lymphoma Treated with Da-Epoch-R. Blood (2021). | results from same studies |
| Zhang XY. Hiv Status Does Not Impact on the Outcome of Patients with Burkitt Lymphoma: A Uk Analysis. British journal of haematology (2021). | results from same studies |
| Euctr IT. Trial on Safety and Activity of Intensive Short-Term Chemoimmunotherapy in Patients with Aids Affected by Burkitt's Lymphoma. https://trialsearchwhoint/Trial2aspx?TrialID=EUCTR2011-003487-75-IT (2011). | data not extractable |
| Ribrag V. Addition of Rituximab Improves Outcome of Hiv Negative Patients with Burkitt Lymphoma Treated with the Lmba Protocol: Results of the Randomized Intergroup (Graall-Lysa) Lmba02 Protocol. (Igr Sponsored Lmba02, Nct00180882). Blood. | data not extractable |
| Jprn U. Phase Ii Study of Multi-Agent Chemotherapy in Patients with Adult Burkitt Leukemia. https://trialsearchwhoint/Trial2aspx?TrialID=JPRN-UMIN000010641 (2013). | data not extractable |
| Jprn U. Phase Ii Study of the Addition of Rituximab to Dmcodox-M/Ivac Therapy for Aids-Related Burkitt Lymphoma. https://trialsearchwhoint /Trial2aspx?TrialID=JPRN-UMIN000011661 (2013). | data not extractable |
| Alwan F. The Addition of Rituximab to Codox-M/Ivac Chemotherapy in the Treatment of Burkitt Lymphoma Is Safe and Is Associated with Increased Efficacy in the Hiv Positive Population. Haematologica (2014). | data not extractable |
| Euctr NL. Phase Iii Study Comparing R-Codox-M/R-Ivac Versus Dose-Adjusted Epoch-R (Da-Epoch-R) for Patients with Newly Diagnosed High Risk Burkitt Lymphoma. https://trialsearchwhoint/Trial2aspx? Trial ID=EUCTR2013-004394-27-NL (2014). | data not extractable |
| Smyth L. Burkitt Leukaemia/Lymphoma: R-Codox-M/R-Ivac Remains Gold Standard Treatment in Bl. Irish Journal of Medical Science (2016). | data not extractable |
| Euctr GB. A Clinical Study Comparing Two Different Chemotherapy Options for Patients with Newly Diagnosed Burkitt Lymphoma. https://trialsearchwhoint/Trial2aspx?TrialID=EUCTR2013-004394-27-GB (2018). | data not extractable |
| Maruyama D. Modified cyclophosphamide, vincristine, doxorubicin, and methotrexate (CODOX-M)/ifosfamide, etoposide, and cytarabine (IVAC) therapy with or without rituximab in Japanese adult patients with Burkitt lymphoma (BL) and B cell lymphoma, unclassifiable, with features intermediate between diffuse large B cell lymphoma and BL. | case report and included patients≤10 |
| Song JY. Burkitt Leukemia Limited to the Bone Marrow Has a Better Prognosis Than Burkitt Lymphoma with Bone Marrow Involvement in Adults. Leukemia & lymphoma (2016). | case report and included patients≤10 |
| Vilaça A. Intracardiac Mass from Burkitt's Lymphoma in an Immunocompromised Patient: A Very Rare Form of Presentation. BMJ case reports (2017). | case report and included patients≤10 |
| Jindal N. Dose-Adjusted Epoch-R: A Feasible Alternative for Burkitt Lymphoma in Resource-Constrained Settings. British journal of haematology (2020). | case report and included patients≤10 |
| Takayama Y. Intestinal Perforation Secondary to Intestinal Burkitt Lymphoma. International Journal of Surgery Case Reports (2021). | case report and included patients≤10 |
| Mead GM. A Prospective Clinicopathologic Study of Dose-Modified Codox-M/Ivac in Patients with Sporadic Burkitt Lymphoma Defined Using Cytogenetic and Immunophenotypic Criteria (Mrc/Ncri Ly10 Trial). Blood (2008). | multiple population |
| Wasterlid T. Impact of Chemotherapy Regimen and Rituximab in Adult Burkitt Lymphoma: A Retrospective Population-Based Study from the Nordic Lymphoma Group. Annals of Oncology (2013). | multiple population |
| da Silva WF. Treatment Outcomes of Adult Burkitt Lymphoma: Results with a Modified Lmb Protocol in Brazil and Feasibility of Outpatient Administration. Journal of Chemotherapy (2018). | multiple population |
| Matsuda S. Is Da-Epoch -/+ Rituximab for Aggressive Lymphoma Feasible and Effective in Clinical Practice? A Retrospective Study. HemaSphere (2018). | multiple population |
| Oosten LEM. Treatment of Sporadic Burkitt Lymphoma in Adults, a Retrospective Comparison of Four Treatment Regimens. Annals of hematology (2018). | multiple population |
| Patekar M. Adult Burkitt Lymphoma: An Institutional Experience with a Uniform Chemotherapy Protocol. South Asian journal of cancer (2018). | multiple population |
| Yang D. Long Term Efficacy of Copadm Regimen in the Treatment of 20 Patients with Burkitt Lymphoma. Zhonghua xue ye xue za zhi = Zhonghua xueyexue zazhi (2018). | multiple population |
| Tan L. Effect of Modified Lmb 89+/- Rituximab Regimen on Long-Term Benefits for Patients's with Burkitt Lymphoma. Zhongguo shi yan xue ye xue za zhi [Journal of experimental hematology] (2020). | multiple population |
