# Supplementary figures and images for: Comparison of chemotherapy regimens plus rituximab in adult Burkitt lymphoma: A single-arm meta-analysis
Source: Front Oncol. 2022 Dec 23;12:1063689. doi: 10.3389/fonc.2022.1063689 (PMC9816660; doi:10.3389/fonc.2022.1063689)

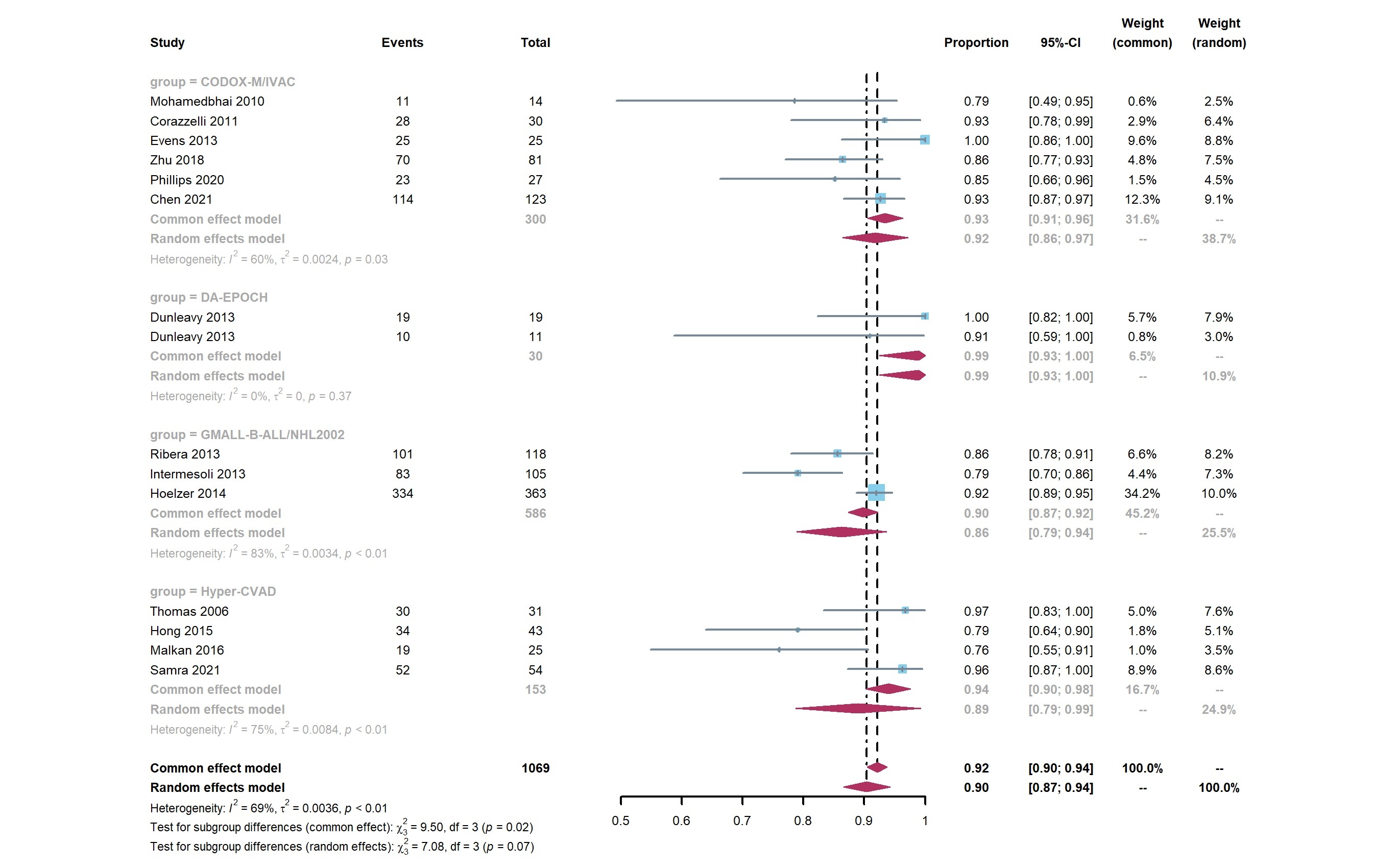

Supplement: Supplementary Figure 1 — Pooled ORR according to regimen group [file Image_1.jpeg]

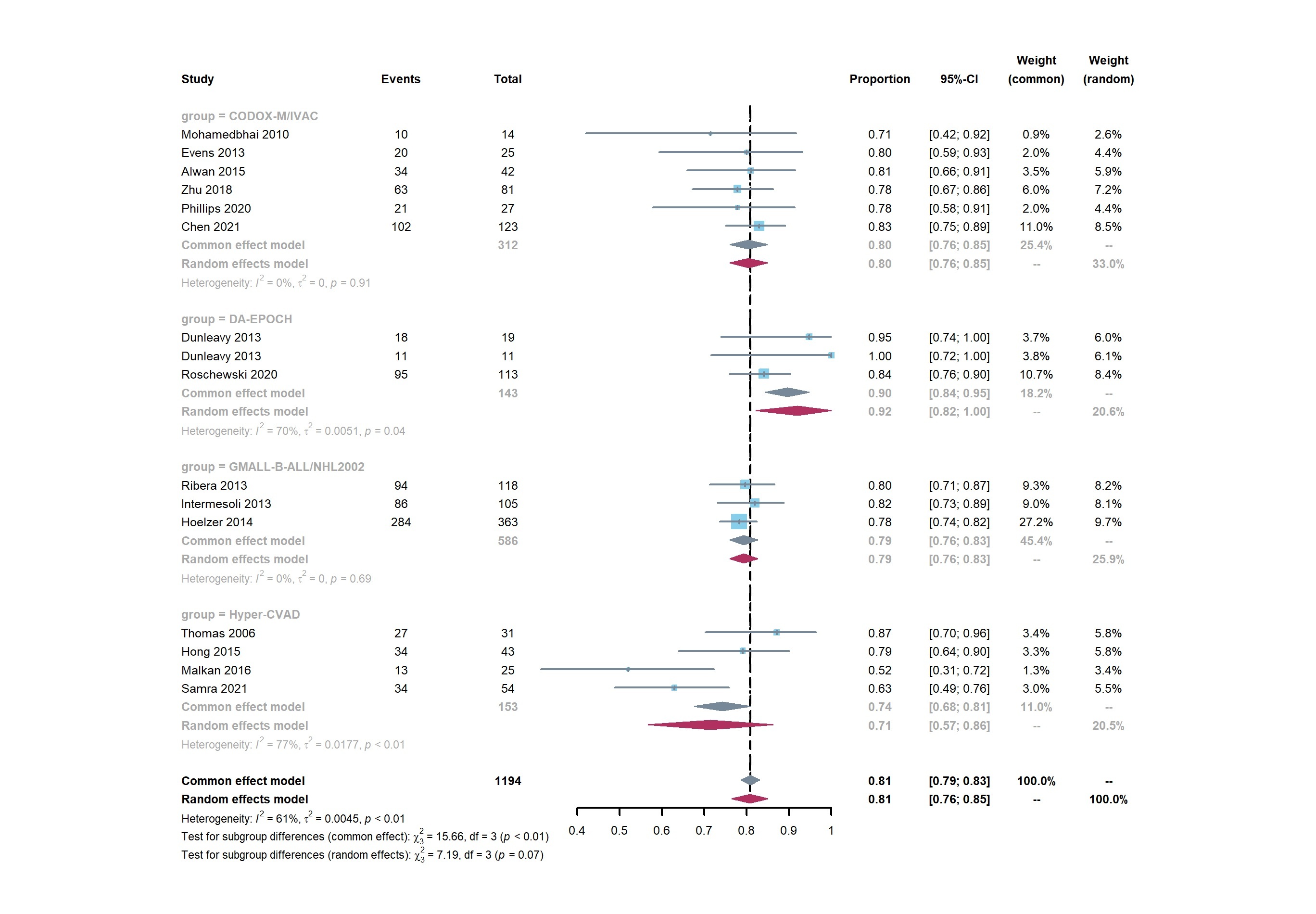

Supplement: Supplementary Figure 2 — Pooled 2-year PFS rate according to regimen group [file Image_2.jpeg]

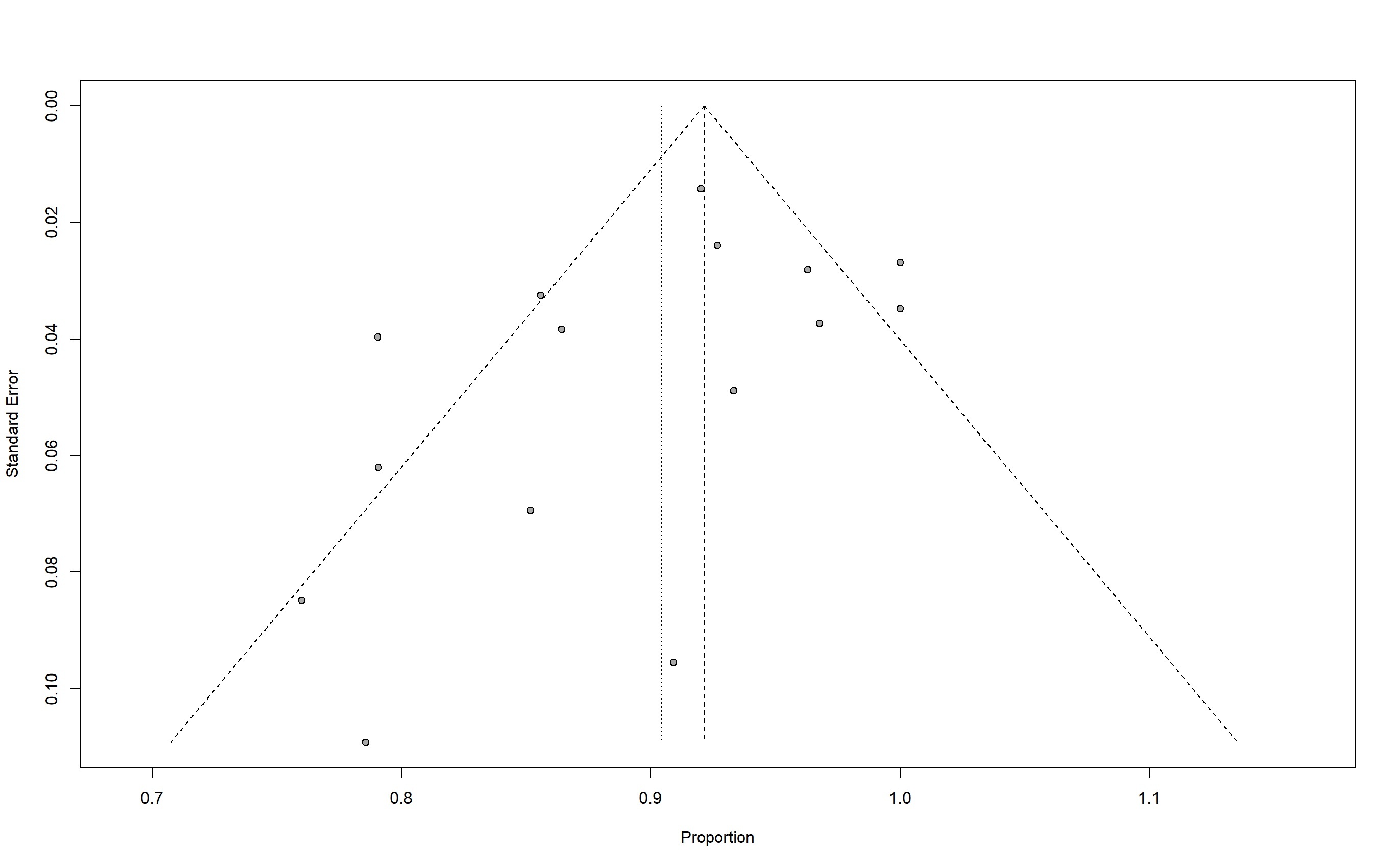

Supplement: Supplementary Figure 3 — Funnel plot of ORR [file Image_3.jpeg]

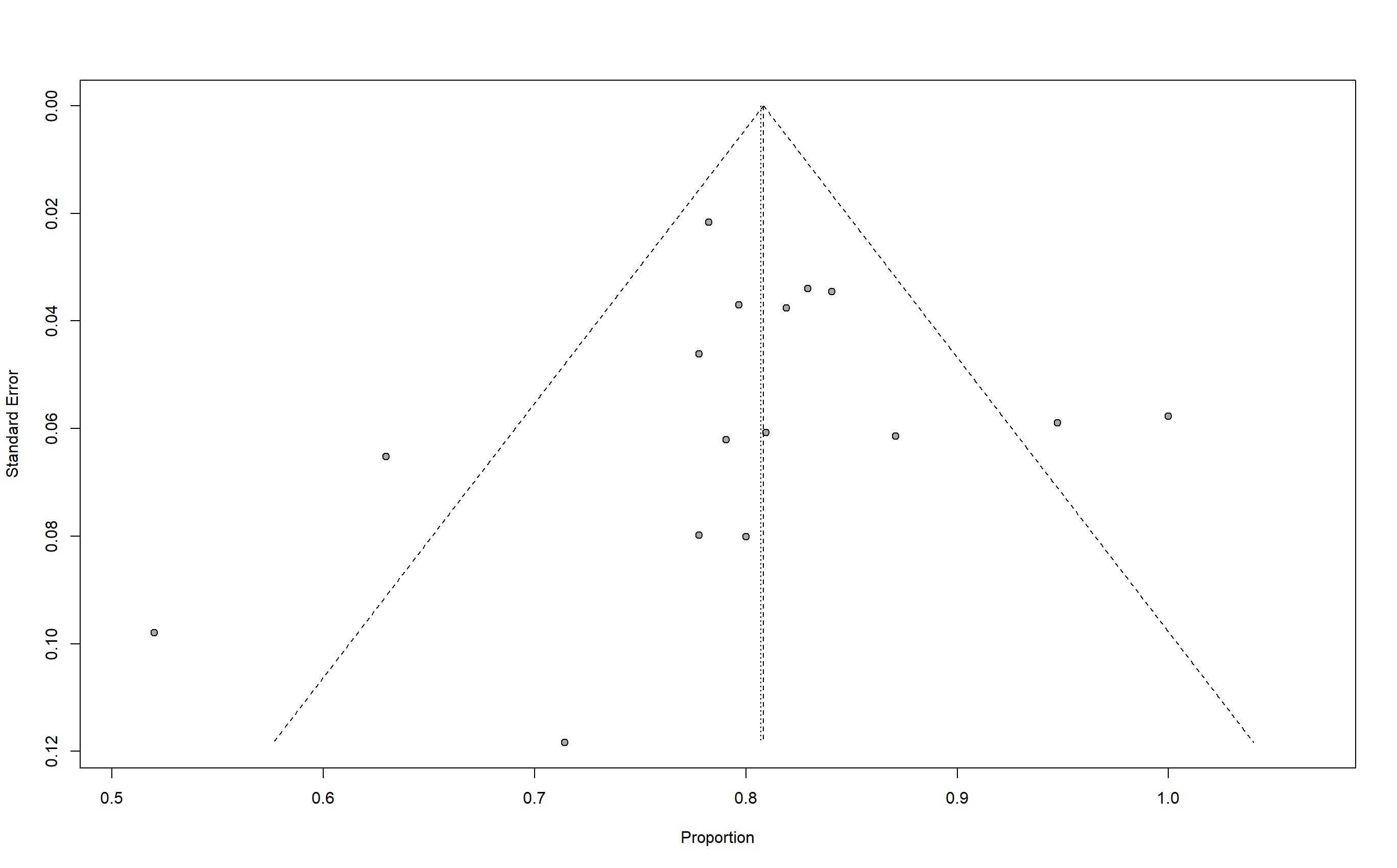

Supplement: Supplementary Figure 4 — Funnel plot of 2-year PFS rate [file Image_4.jpeg]
